# Mpox alert: COVID-19's hard-earned lessons put to the test

**DOI:** 10.1016/j.onehlt.2025.101003

**Published:** 2025-02-25

**Authors:** Babatope O. Adebiyi

**Affiliations:** Section of Rheumatology, Department of Paediatrics, University of Calgary, Calgary, Alberta, Canada

**Keywords:** Mpox outbreak, Clade Ib, Public health emergency, Global Health, Surveillance systems, Vaccine equity, Community engagement, Infectious disease response, COVID-19 lessons

## 1 Introduction

The current mpox outbreak represents a significant global health challenge, with the World Health Organization declaring it a public health emergency of international concern on August 14, 2024 [1]. This declaration followed a sharp increase in mpox cases in the Democratic Republic of the Congo (DRC) and its spread to neighboring countries such as Kenya, Rwanda, Burundi, Republic of Congo, Central African Republic, Uganda, Zambia, and Zimbabwe. The outbreak is characterized by the emergence of a new strain of the virus, known as Clade Ib, which is affecting previously unaffected areas [1]. This new strain (Clade Ib) emerged in the DRC's South Kivu province around September 2023 and is part of Clade I. Early evidence suggests that it is more common among youth and adults 15 years and older and may be less severe but potentially more transmissible than Clade Ia [1]. Concurrently, there has been an increase in Clade Ia cases in the DRC, Central African Republic, and Republic of Congo [1].

This outbreak follows a previous global mpox event that began in May 2022, notable for being the largest documented outbreak of mpox in terms of magnitude and geographical spread [1]. That event primarily affected non-endemic countries and saw transmission occurring mainly through direct and intimate contact, particularly among men who have sex with men. The current outbreak presents new challenges with its rapid spread in endemic regions and the emergence of a potentially more transmissible strain.

This situation underscores the ongoing challenge of mpox and highlights the need for continued vigilance, research, and global cooperation in addressing emerging infectious diseases. It also emphasizes the importance of applying lessons learned from previous outbreaks, including the COVID-19 pandemic, to improve response strategies and preparedness for future health emergencies [2].

This opinion piece explores how critical lessons from the COVID-19 pandemic—such as the importance of global surveillance, equitable vaccine distribution, rapid containment strategies, and community engagement—can inform and enhance the response to the current mpox outbreak. Both outbreaks share key characteristics as global health emergencies requiring rapid, coordinated international action to prevent widespread impact. COVID-19 demonstrated the devastating consequences of delayed responses, inequitable access to healthcare resources, and insufficient public health communication. Applying these lessons to mpox is vital to improving outbreak management, mitigating transmission, and strengthening preparedness for future global health crises.

## 2 Application of COVID-19 lesson

Applying lessons learned from the COVID-19 pandemic is crucial for an effective response to mpox. These lessons include:

### 2.1 Strengthening surveillance and rapid response

Early detection and rapid response are essential for controlling outbreaks. The COVID-19 pandemic underscored the importance of effective surveillance systems for quickly identifying and reporting cases. For instance, countries such as South Korea, which implemented comprehensive testing and contact-tracing, were able to limit the virus's spread [3]. A similar approach for mpox could help isolate cases and prevent widespread transmission. To achieve this, robust global surveillance networks are crucial. These systems should focus on strengthening laboratory capacity for quick diagnostic testing, including clade-specific tests, especially in regions with high international travel. Additionally, creating centralized platforms for real-time data sharing is necessary to track trends and pinpoint hotspots.

### 2.2 Ensuring vaccine equity and resource distribution

The COVID-19 pandemic highlighted disparities in vaccine distribution, with low-income countries facing significant delays despite initiatives like COVAX (COVID-19 vaccines global access) [4]. For mpox, equitable distribution can be achieved by decentralizing production to encourage local manufacturing of vaccines and diagnostics, reducing reliance on external suppliers. Additionally, resources must be allocated to ensure timely access for low- and middle-income countries, drawing on lessons from COVAX's successes and shortcomings (while COVAX delivered over a billion vaccine doses globally, logistical challenges and unequal prioritization led to slower rollouts in Africa).

### 2.3 Community engagement

Community engagement is crucial in managing public health crises, as demonstrated during COVID-19, where misinformation about vaccines and treatments hindered response efforts. Initiatives like UNICEF's community-based programs in low-resource settings highlighted the importance of local partnerships in effectively spreading accurate health information and improving adherence to preventive measures [5]. For mpox, engaging community leaders, faith-based organizations, and local health workers is essential to tailor health messages that resonate with cultural practices. Additionally, investing in clear and consistent communication strategies, including using social media and grassroots campaigns, can help combat misinformation and ensure the public receives accurate and timely information.

### 2.4 Enhancing international collaboration

International collaboration was critical during the COVID-19 pandemic, particularly in vaccine development and distribution, with initiatives like the GAVI Alliance and CEPI ensuring global access [6]. The rapid development of mRNA vaccines by Pfizer-BioNTech and Moderna exemplified the power of unprecedented global cooperation. For mpox, similar collaboration is essential. Promoting data sharing, including case numbers, genomic sequencing, and vaccine efficacy outcomes, will help track and respond to the outbreak effectively. Streamlining regulatory processes for faster cross-border vaccine access and fostering global research partnerships will be crucial for accelerating the development of mpox vaccines and treatments, mirroring the successful models of COVID-19 vaccine development.

## 3 Potential barriers to implementing lessons from COVID-19 for Mpox

Implementing lessons from COVID-19 to address the mpox outbreak faces several barriers. One major challenge is the stigma associated with mpox, particularly because it has disproportionately affected certain groups, such as men who have sex with men. This stigma discourages individuals from seeking testing or treatment and may exacerbate misinformation about the disease. To counter this, public health campaigns must adopt inclusive and non-stigmatizing messaging that emphasizes the universal risk of infection. Engaging trusted community leaders can help disseminate accurate information and promote solidarity over blame.

Resource limitations in endemic regions also hinder effective responses. Many affected countries lack the infrastructure for robust surveillance, testing, and vaccination efforts. Strengthening international support through funding mechanisms like an adapted COVAX model and encouraging regional vaccine production can help address these gaps. Providing technical assistance and training for healthcare workers in low-resource settings will further enhance local capacity to respond effectively.

Another barrier is inconsistent data sharing, which complicates coordinated global efforts. Timely reporting of case numbers, genomic sequencing, and vaccine efficacy is critical but often delayed, particularly in resource-limited regions. Establishing global surveillance networks and supporting countries in developing efficient data systems can improve transparency and collaboration.

Equitable vaccine distribution and hesitancy also remain challenges. Wealthier nations often secure vaccines at the expense of low-income countries, while misinformation and cultural factors contribute to hesitancy. Addressing these issues requires decentralizing vaccine production, ensuring affordable access, and implementing culturally sensitive education campaigns to build public trust in vaccines.

Finally, engaging communities effectively can be difficult, particularly in areas with historical distrust of health authorities. Partnering with community-based organizations and local health workers can foster trust and deliver tailored, culturally relevant health messages. By addressing these barriers with inclusive and coordinated strategies, the lessons from COVID-19 can be effectively applied to mitigate the mpox outbreak.

## 4 Conclusion

The mpox outbreak is more than a health crisis—it is a pivotal opportunity to demonstrate the global community's commitment to learning from past pandemics. The strategies honed during COVID-19, such as early detection, equitable resource distribution, and robust community engagement, must now be translated into action. However, this requires urgency and collective resolve. Governments, international organizations, and local communities must collaborate to strengthen surveillance systems, ensure fair access to vaccines and treatments, and empower grassroots initiatives to build trust and resilience. The stakes are high. The emergence of infectious diseases like mpox underscores the interconnectedness of global health, where the vulnerabilities of one region can quickly become a worldwide concern. The world cannot afford complacency. By uniting in decisive and coordinated action, we can not only control the current outbreak but also create a stronger foundation for responding to future health emergencies. Now is the time to act—swiftly, inclusively, and collaboratively—to safeguard global health and equity.

## Consent to publish

Not applicable.

## Funding

Not applicable.

## Declaration of generative AI and AI-assisted technologies in the writing process

During the preparation of this work the author used ChatGPT to enhance the structure and grammar of the manuscript. After using this tool/service, the author reviewed and edited the content as needed and takes full responsibility for the content of the publication.

## CRediT authorship contribution statement

**Babatope O. Adebiyi:** Writing – review & editing, Writing – original draft, Visualization, Validation, Supervision, Software, Resources, Project administration, Methodology, Investigation, Funding acquisition, Formal analysis, Data curation, Conceptualization.

## Declaration of competing interest

The author declares no conflicts of interest relevant to this work.

## Data Availability

No data was used for the research described in the article.
